# Assessment of the Utility of the Septal *E*/(*E*′ × *S*′) Ratio and Tissue Doppler Index in Predicting Left Ventricular Remodeling after Acute Myocardial Infarction

**DOI:** 10.1155/2016/4954731

**Published:** 2016-09-15

**Authors:** Selma Kenar Tiryakioglu, Hakan Ozkan, Hasan Ari, Kıvanc Yalin, Senol Coskun, Osman Tiryakioglu

**Affiliations:** ^1^Bursa State Hospital, Department of Cardiology, Bursa, Turkey; ^2^Department of Cardiology, Faculty of Medicine, Bahcesehir University, Istanbul, Turkey; ^3^Bursa Yuksek Ihtisas Education and Research Hospital, Department of Cardiology, Bursa, Turkey; ^4^Department of Cardiovascular Surgery, Faculty of Medicine, Bahcesehir University, 16110 Istanbul, Turkey

## Abstract

*Background*. The aim of this study is to show whether the septal *E*/(*E*′ × *S*′) ratio assessed by tissue Doppler echocardiography can predict left ventricular remodeling after first ST segment elevation myocardial infarction treated successfully with primary percutaneous intervention.* Methods*. Consecutive patients (*n* = 111) presenting with acute anterior myocardial infarction for the first time in their life were enrolled. All patients underwent successful primary percutaneous coronary intervention. Standard and tissue Doppler echocardiography were performed in the first 24-36 hours of admission. Echocardiographic examination was repeated after 6 months to reassess left ventricular volumes. Septal *E*/(*E*′ × *S*′) ratio was assessed by pulsed Doppler echocardiography.* Results*. Group 1 consisted of 33 patients with left ventricular (LV) remodeling, and Group 2 had 78 patients without LV remodeling. *E*/(*E*′ × *S*′) was significantly higher in Group 1 (4.1 ± 1.9 versus 1.65 ± 1.32, *p* = 0.001). The optimal cutoff value for *E*/(*E*′ × *S*′) ratio was 2.34 with 87.0% sensitivity and 82.1% specificity.* Conclusion*. Septal *E*/(*E*′ × *S*′) values measured after the acute anterior myocardial infarction can strongly predict LV remodeling in the 6-month follow-up. In the risk assessment, the septal *E*/(*E*′ × *S*′) can be evaluated together with the conventional echocardiographic techniques.

## 1. Introduction

Acute myocardial infarction (AMI) is a sudden onset condition and is often fatal. The ventricular geometry changes in the infarct related area after AMI [1, 2]. The change in ventricular geometry is defined as remodeling that is directly related to prognosis [1, 2]. Percutaneous interventional therapy is the gold standard treatment in acute myocardial infarction; however ventricular remodeling is seen in only 30% of patients complicated with AMI [3]. Therefore, early identification of patients at risk of developing LV remodeling after AMI has important prognostic and therapeutic implications [4]. Echocardiographic evaluation is a routine procedure for the risk assessment. Left ventricular ejection fraction is an important parameter for the evaluation of systolic function, and diastolic dysfunction parameters are independent predictors of mortality in these patients. Doppler tissue imaging (DTI) echocardiography is already a part of standardized diastolic evaluation. The pulsed Doppler TDI parameters are at the forefront of clinical usage due to previous studies. Early diastolic transmitral velocity/early mitral annular velocity ratio (*E*/*E*′) is one of the most well-known evaluation methods. The *E*/*E*′ ratio has been proposed to be the single best Doppler predictor for evaluating LV filling pressure and is a good predictor of cardiac death [5].

Mornos et al. studied an index including the *S* value that is a systolic parameter in 2009 and found that this index [*E*/(*E*′ × *S*′)] predicts left ventricular (LV) filling pressure in different populations [6]. In another study published in 2014, Mornos et al. declared the predictive value of this index in patients with heart failure [7]. We investigated the usefulness of this index in patients with acute anterior myocardial infarction who were successfully treated with percutaneous interventional therapy according to these two clinical studies. We investigated the predictive value of this index in patients with and without positive left ventricle remodeling.

## 2. Methods

### 2.1. Study Population

This study was designed as a prospective study. One hundred and eleven patients with acute anterior myocardial infarction enrolled in the study between January 2014 and December 2015. Written, informed consent was taken from all patients. We obtained approval from local ethical committee. Fourteen patients were excluded due to poor echocardiographic assessment. Acute myocardial infarction was defined as >2 mm ST elevation in precordial leads or new onset LBBB in electrocardiography in patients with a history of 30 minutes to 12 hours of chest pain. The diagnosis was confirmed with troponin levels. Troponin T (Tn T), creatine kinase (CK), and MB fraction of CK (CK-MB) were measured at baseline and at the end of 6th and 24th hours. The major exclusion criteria were a history of other heart muscle and valvular diseases (e.g., heart failure, cardiomyopathies, significant valvular defects, congenital defects, and myocarditis), systemic diseases affecting left ventricular systolic and diastolic functions, serious arrhythmias including atrial fibrillation, permanent pacemaker implantation history, and patients with poor acoustic window in echocardiography. According to these criteria, 111 patients were enrolled and 24 patients were excluded. Patients were divided into two groups according the remodeling at the end of the 6th month.

### 2.2. Echocardiographic Study

Echocardiography was performed with a Vivid 3 ultrasound system (GE Vingmed Ultrasound AS, Horten, Norway) by cardiologist between 24 and 36 hours after primary PCI. All participants were examined using conventional two-dimensional echocardiography and pulsed-wave Doppler tissue imaging (DTI) according to standardized protocols. All echocardiograms were stored and analyzed by two cardiologists. The LV dimensions (septum, posterior wall, LV end diastolic and end systolic diameter) were obtained from parasternal long axis view. The pulsed-wave sample volume is placed between the tips of the mitral leaflets ensuring parallel alignment of the Doppler beam with blood flow. Peak velocities of early (*E*) and atrial (*A*) diastolic filling and *E* wave deceleration (DT) were measured, and the* E/A* ratio was calculated. The LV ejection fraction (LVEF) and LV volumes were determined using modified biplane Simpson's method. All measurements used for statistical analysis were averaged from five consecutive beats. The LV was divided into 17 segments, evaluated, and scored (1: normal, 2: hypokinesis, 3: akinesis, 4: dyskinesis, and 5: aneurysmal). The global wall motion score index (WMSI) was calculated for each TTE examination. More than 20% increase in the diastolic volume and/or systolic volume was defined as left ventricular remodeling [3].

Transmitral flow patterns were recorded from apical four-chamber windows with 4-5 mm pulsed-sample Doppler volume placed between mitral valve tips in diastole during five consecutive cardiac cycles. Care was taken to obtain the smallest possible angle between the direction of transmitral flow and the ultrasound beam. The measurements were recorded during the expiratory apnea and the mean value of five consecutive measurements obtained. Pulsed Doppler signals were recorded at a horizontal sweep of 100 mm/s. The TDI program was set in pulsed-wave Doppler mode. Motion of mitral annulus was recorded in the apical four-chamber view at a frame rate of 80 to 140 frames per second [8]. A 4-5 mm sample volume was positioned sequentially at the lateral and septal corners of the mitral annular velocity (*E*′), and peak mitral annular systolic velocity (*S*′) was measured. The *E*/*E*′ and *E*/(*E*′ × *S*′) values were recorded from septal and lateral mitral annulus [8]. Similarly, the measurements were recorded during the expiratory apnea and the mean value of five consecutive measurements was obtained for analysis. All measurements were repeated at the end of the 6th month.

### 2.3. Clinical Variables

Baseline characteristics including gender, age, hypertension, diabetes mellitus, hyperlipidemia, smoking, family history, and body mass index of the patients who presented with acute myocardial infarction were evaluated. In addition, blood urea, creatinine, uric acid, high density lipoprotein (HDL) and low density lipoprotein (LDL) cholesterol, troponin peak CK-MB and hemoglobin, platelet count (PLT), and neutrophil-leucocyte levels were analyzed. Angiographic parameters including multivessel disease, procedures for the treatment (PTCA, PTCA + stent, and direct stenting), antiplatelet therapy, and TIMI flow grade before and after procedure were recorded.

### 2.4. Primary PCI Procedure

Primary PCI procedures were obtained according to international guidelines. 300 mg chewable aspirin as well as clopidogrel, prasugrel, or ticagrelor was given with certain loading doses. 10000 U unfractionated heparin was used during the procedure. GPIIb/IIIa antagonist agents were used if necessary. Multivessel disease is defined as two- or three-vessel disease with complexity of the lesions. Anti-ischemic, antiplatelet, and lipid lowering therapy after the procedure was given and continued according to guidelines. Two invasive cardiologists assessed the coronary angiograms. TIMI thrombus grade scales were evaluated and patients with high thrombus scale were identified [9, 10]. Syntax score was assessed with an online calculator, and corrected TIMI frame counts were defined [11–13].

### 2.5. Clinical Outcome

All echocardiographic measurements including TDI parameters were recorded at the end of the 6th month, and patients were divided into two groups according to left ventricular remodeling using the left ventricular systolic and diastolic volumes.

### 2.6. Statistical Analysis

Measured values are reported as the mean ± standard deviation, and statistical comparisons were performed using SPSS11.0. The normality of the distribution of the groups was compared using a Kolmogorov-Smirnov test, and categorical variables were analyzed using a chi-square test. A *t*-test was used for the comparison of echocardiographic parameters between two groups. Receiver operating characteristic analysis (ROC) was used for the determination of predictive value. A *p* value <0.05 was considered significant.

## 3. Results

Hundred and eleven patients (58 ± 14.8 years, 27 women) were included in the study at the end of the 6th month. Group 1 consisted of 33 (29.7%) patients with positive remodeling, and the rest of the patients without remodeling were defined as Group 2. Baseline characteristics of the study population are shown in Table 1. The baseline characteristics were similar between the two groups, but the prevalence of diabetes mellitus was higher in Group 2 (*p* = 0.016).

The pain-balloon time was high in the remodeling group when the groups were compared according to clinical characteristics. However the difference was not statistically significant. Similarly, the comparison of two groups according to syntax score and TIMI thrombus scale was not significant. Peak troponin and CK-MB levels were similar between the two groups despite the higher values in Group 1 (Table 2).

Baseline and the end of the 6th month measurements of echocardiographic data including TDI values were compared between the two groups. The comparison of mitral *E* velocity and the* E/A* ratio between two groups was similar. Septal *E*′ and *S*′ values in Group 1 were significantly lower than Group 2. The septal *E*/*E*′ ratio was significantly higher in patients with positive remodeling (16.4 ± 5.9 versus 10.1 ± 2.64, *p* = 0.003). Similarly, *E*/(*E*′ × *S*′) was significantly higher in Group 1 (4.1 ± 1.9 versus 1.65 ± 1.32, *p* = 0.001). The initial LVEF was lower (35.7 ± 4.8 versus 42.4 ± 6.5, *p* = 0.001) when WMSI was higher (1.99 ± 0.42 versus 1.57 ± 0.42, *p* = 0.04) in patients with positive remodeling. The end baseline diastolic and systolic volume indexes in Group 1 were high but not statistically significant. On 6-month follow-up, the EDVI was significantly higher in Group 1 (91 ± 20.5 versus 61.4 ± 13.1, *p* = 0.001) and ESVI was higher in Group 1 as well (50.3 ± 16.4 versus 38.4 ± 9.8, *p* = 0.011). In the first 24–36 hours of admission, the deceleration time (DT) and isovolumetric relaxation time (IVRT) were shorter in Group 1 but not significant (Table 3).

**Figure 1:**
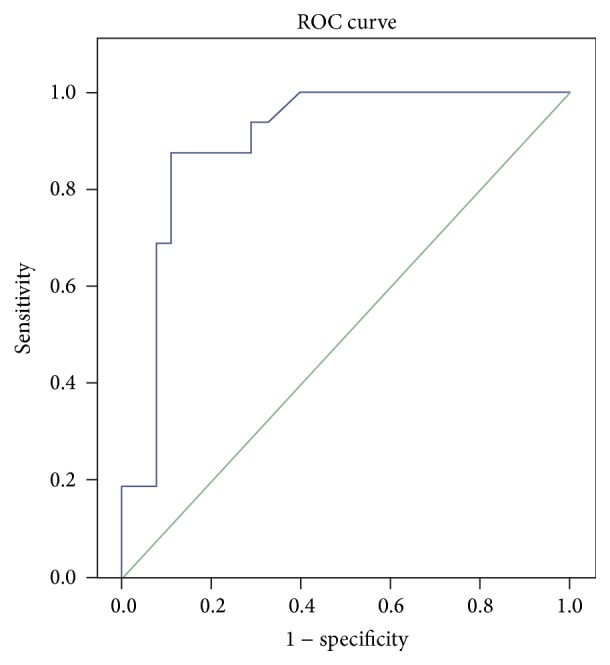
Receiver operating characteristic analysis (ROC). Diagonal segments are produced by ties.

Figure 1 is a ROC curve for *E*/(*E*′ × *S*′) at discharge to predict remodeling. The optimal cutoff value for *E*/(*E*′ × *S*′) ratio was 2.34 with 87.0% sensitivity and 82.1% specificity (Figure 1).

## 4. Discussion

Echocardiography has an important place in the assessment of left ventricular functions after myocardial infarction. Echocardiographic parameters indicating systolic and diastolic dysfunction have been studied for many years. In patients after acute myocardial infarction, the restrictive filling pattern or the shortened deceleration time predicts LV remodeling and mortality [5, 14]. Hillis et al. evaluated 73 of 250 patients (29%) with *E*/*E*′ > 15 and demonstrated that the *E*/*E*′ ratio was a strong predictor [14]. This study showed that the *E*/*E*′ ratio is a better predictor than the other clinical and echocardiographic parameters. Hillis et al. evaluated the echocardiographic parameters of 47 patients with acute myocardial infarction at the end of the 3rd day and 8th week in 2006. *E*/*E*′ was much higher in patients who had increased LV end diastolic volume >15% versus the value in patients without remodeling. According to this study, the *E*/*E*′ ratio can predict LV remodeling associated with LV dilatation [15]. Currently, the *E*/*E*′ ratio is one of the most commonly used echocardiographic parameters in clinical practice [16]. Mitral annular velocities reflect the long axis motion of the ventricle, which is an important component of LV systolic and diastolic function.

In a study that evaluated *E*′, *S*′, and *A*′ values for the prediction of the cardiac mortality, *S*′ has been shown to be a good measurement of global systolic function [17]. Wang et al. found that *S*′ wave may determine early systolic impairment in patients with diastolic dysfunction and preserved LVEF [18]. Similarly, the prognostic value of *E*′ velocity was found to be strong in the same study. Another study from 2012 compared the pulsed-wave *S*′ value in patients with and without diastolic dysfunction and demonstrated the importance of the *S*′ value to show early impairment of the longitudinal systolic functions. The impairment of the systolic dysfunction started before the deterioration of the diastolic dysfunction independently [19].

Mornos et al. demonstrated that the *E*/(*E*′ × *S*′) value predicted the cardiac mortality in patients with heart failure. In this study, Mornos et al.thought that the *E*/*E*′ ratio was insufficient for the prediction of the LV end diastolic filling pressure and indicated the restrictions when *E*/*E*′ ratio was between 8 and 15 in healthy individuals. The relationship between systolic performance and diastolic function is not clear. Conceptually, it is very difficult to separate relaxation from contraction, and it is better to consider them together as a part of a continuous cycle [6]. Systolic and diastolic abnormalities affect the LV filling pressure in different levels in conditions such as congestive heart failure. In our study, a new index, *E*/(*E*′ × *S*′) was evaluated and found to be strong predictor in patients with regional wall motion abnormality when compared with *E*/*E*′ ratio [6]. Here, we evaluated the effect of this index on the positive remodeling that can be measured more easily than the other echocardiographic parameters. *E*/(*E*′ × *S*′) was found to be an important index that evaluates both the diastolic and systolic parameters via a systolic parameter *S*′ value.

Myocardial infarction causes complex changes in the infarct related area. This change is defined as ventricular remodeling and affects the ventricular functions and prognosis. Remodeling after myocardial infarction is related to adverse cardiac events and mortality. The maintenance of the normal flow in the infarct related artery reduces the ventricular remodeling even in the late period. However, remodeling may be seen even after successful primary coronary intervention [20]. We aimed to investigate the utility of this index for the prediction of remodeling in patients with acute anterior myocardial infarction who were successfully treated with percutaneous interventional therapy according to this idea.

We found remodeling in 29.7% of the patients successfully treated with primary PCI. The *E*/(*E*′ × *S*′) index after primary PCI was 87% sensitive and 81.2% specific. The *E*/(*E*′ × *S*′) index can be easily measured in echocardiographic examination and will have important usage because the *E*/*E*′ ratio is commonly used in clinical practice.

Biering-Sørensen et al. investigated the color Doppler parameters in patients treated with primary PCI and found the *S*′ and *E*′ values predict adverse cardiac events in a study published in 2014 [20]. Similarly *E*/(*E*′ × *S*′) index predicts ventricular remodeling in our study. We considered the successful usage of advance Doppler echocardiographic parameters for the prediction of ventricular remodeling in the literature [21]. However, *E*/(*E*′ × *S*′) index is easier and more practical for predicting remodeling—this is what differentiates our study from the others. *E*/(*E*′ × *S*′) values >2.34 can be used in the clinical practice according to ROC curve analysis.

## 5. Limitations

The study consisted of a small number of patients and was statistically significant. We did not use advanced Doppler parameters because they are not useful in clinical practice.

The invasive parameters were not compared in this study. This is another limitation of the study. Mornos et al. proved the correlation of this parameter with invasive end diastolic volumes of the measurements in their study. Therefore, we cited this article as a reference and we did not want to prove it again. Another limitation of the study is that Mornos et al.'s study has been the only study done on that. Finally, our study is a single center study, and its reproduction in other centers or by multicenter studies could further confirm its validity.

## 6. Conclusion

The *E*/(*E*′ × *S*′) index may predict left ventricular remodeling at the end of the 6th month in patients successfully treated with primary PCI. *E*/(*E*′ × *S*′) value >2.34 predicts positive remodeling independent of LVEF.

## Figures and Tables

**Table 1:** Baseline characteristics.

	Group 1 (*n* = 33)	Group 2 (*n* = 78)	*p* value
Age	59.1 ± 18.4	57.3 ± 12.6	NS
Female	9 (27%)	18 (30%)	NS
Hypertension	14 (42.2%)	27 (34.6%)	NS
Diabetes mellitus	12 (36%)	18 (23%)	0.016
Smoking	15 (45%)	30 (38.4%)	NS
Family history	21 (63%)	39 (50%)	NS
BMI	26.0 ± 3.6	27.9 ± 4.4	NS

BMI: body mass index. *p* > 0.05 nonsignificant.

**Table 2:** Clinical characteristics.

	Group 1 (*n* = 33)	Group 2 (*n* = 78)	*p* value
Peak troponin (ng/mL)	7.62 ± 4.8	6.5 ± 4.1	NS
Peak CK-MB (ng/mL)	227.6 ± 168.7	203.3 ± 176.8	NS
TIMI thrombus score	3.8 ± 1.1	3.2 ± 1.2	NS
SYNTAX score	16.6 ± 6.6	19.5 ± 7.4	NS
Pain-balloon time	263.75 ± 56.40	229.25 ± 63.39	NS
cTFC	25.0 ± 5.77	22.22 ± 4.6	NS

cTFC: corrected TIMI frame count, NS: nonsignificant, and TIMI: thrombolysis in myocardial infarction (*p* > 0.05 nonsignificant).

**Table 3:** Comparison of two groups according to echocardiographic parameters.

	Group 1 (*n* = 39)	Group 2 (*n* = 96 )	*p* value
LVDD (cm)	5.1 ± 0.8	4.8 ± 0.3	NS
LVSD (cm)	3.5 ± 1.1	3.3 ± 0.6	NS
LV ejection fraction (%)	35.7 ± 4.8	42.4 ± 6.5	0.001
WMSI	1.99 ± 0.42	1.57 ± 0.42	0.04
LVEDVI mL/m^2^ (baseline)	72 ± 22.4	63 ± 15.1	NS
LVESVI mL/m^2^ (baseline)	38.8 ± 10.5	41.5 ± 9.4	NS
LVEDVI mL/m^2^ (6th month)	91 ± 20.5	61.4 ± 13.1	0.001
LVESDI mL/m^2^ (6th month)	50.3 ± 16.4	38.4 ± 9.8	0.011
*E* (cm/s)	71.0 ± 27.2	72.8 ± 18.9	NS
*E*/*A* ratio	1.6 ± 1.3	1.1 ± 0.5	NS
Deceleration time (ms)	215.2 ± 59	222.3 ± 63	NS
IVRT (ms)	100.9 ± 20.3	107.1 ± 22.3	NS
*E*′ cm/s	5.2 ± 1.3	8.2 ± 2.9	0.001
*S*′ cm/s	4.7 ± 1.4	6.3 ± 1.9	0.007
*E*/*E*′ ratio	16.4 ± 5.9	10.1 ± 2.64	0.003
Sep *E*/(*E*′ × *S*′) ratio	4.1 ± 1.9	1.65 ± 1.32	0.001
Lat *E*/(*E*′ × *S*′) ratio	3.1 ± 1.1	1.3 ± 1.0	0.001

LVDD: left ventricular diastolic diameter, LVSD: left ventricular systolic diameter, WMSI: wall motion score index, LVEDVI: left ventricular end diastolic volume index, LVESVI: left ventricular end systolic volume index, IVRT: isovolumetric relaxation time, and NS: nonsignificant (*p* > 0.05 nonsignificant).
